# Supplementary material for: Association of Anemia with Clinical Symptoms Commonly Attributed to Anemia—Analysis of Two Population-Based Cohorts
Source: J Clin Med. 2023 Jan 24;12(3):921. doi: 10.3390/jcm12030921 (PMC9918126; doi:10.3390/jcm12030921)
Supplement: Supplementary file 1 [file jcm-12-00921-s001.zip › jcm-2042227-supplementary.pdf]

Table S1 Logistic regression models, bivariate analysis. Only estimates for the predictor anemia are shown. (weighted)

Outcomes				
Predictors	Fatigue	Lack of energy	Lack of concentration	Shortness of breath and/or weakness
	OR (95% CI)	OR (95% CI)	OR (95% CI)	OR (95% CI)
Age in years (continuous)	**1.23 (1.02-1.47)**	**1.38 (1.12-1.72**)	1.16 (0.92-1.45)	**1.26 (1.05-1.52)**
Sex				
Male	Reference	Reference	**1.31**	**1.56**
Female	**1.23 (1.02-1.47)**	**1.42 (1.15-1.76)**	**(1.05-1.64)**	**(1.31-1.87)**
Number of medications	1.02 (0.85-1.24)	1.12 (0.89-1.39)	1.02 (0.81-1.28)	1.07 (0.87-1.30)
Medication with side-effects	1.12 (0.93-1.35)	1.23 (0.99-1.53)	1.13 (0.89-1.42)	**1.38 (1.15-1.66)**
Hypothyroidism (claims data)	1.19 (0.99-1.44)	**1.38 (1.11-1.71)**	**1.29 (1.03-1.61)**	**1.55 (1.29-1.85)**
Depression and/or anxiety (claims data)	1.21 (1.00-1.45)	**1.41 (1.14-1.76)**	**1.31 (1.05-1.65)**	**1.57 (1.31-1.88)**
Depression and/or anxiety (SHIP)	**1.25 (1.03-1.51)**	**1.53 (1.21-1.95)**	**1.39 (1.09-1.77)**	**1.61 (1.34-1.94)**
Insomnia (SHIP)	1.15 (0.95-1.39)	**1.32 (1.06-1.65)**	1.24 (0.98-1.55)	**1.51 (1.23-1.81)**
CCI (claims data)	1.10 (0.92-1.33)	**1.25 (1.01-1.56)**	1.12 (0.89-1.41)	1.22 (1.00-1.47)
Cancer (claims data)	1.19 (0.99-1.43)	**1.38 (1.11-1.71)**	**1.29 (1.03-1.62)**	**1.52 (1.27-1.82)**
CKD (claims data)	1.17 (0.98-1.42)	**1.34 (1.08-1.66)**	1.22 (0.97-1.53)	**1.41 (1.18-1.69)**
Heart failure (claims data)	1.17 (0.97-1.41)	**1.34 (1.08-1.66)**	1.24 (0.99-1.55)	**1.41 (1.18-1.70)**
Diabetes (claims data)	1,16 (0,97-1,40)	**1.33 (1.07-1.65)**	1.21 (0.97-1.52)	**1.41 (1.17-1.69)**
Pregnancy	1,20 (1,00-1,44)	**1.38 (1.12-1.71)**	**1.31 (1.04-1.63)**	**1.56 (1.30-1.87)**
COPD (claims data)	-	-	-	**1.48 (1.24-1.78)**
Asthma (claims data)	-	-	-	**1.56 (1.30-1.88)**

OR: Odds Ratio, 95% CI: 95% Confidence Interval, CCI: Charlson comorbidity index,
COPD: Chronic obstructive pulmonary disease, CKD: Chronic kidney disease
bold numbers = statistically significant

Table S2 Multivariate logistic regression models with symptoms of anemia as outcome variables (weighted) including the variable iron deficiency instead of the variable anemia (as compared to **Table 5**)

	Outcomes							
	Fatigue		Lack of energy		Lack of concentration		Dyspnea and/or weakness	
Predictors	OR (95% CI) n = 5777		OR (95% CI) n=5777		OR (95% CI) n=5777		OR (95% CI) n=5777	
	Model 1	Model 2	Model 1	Model 2	Model 1	Model 2	Model 1	Model 2
iron deficiency	1.19 (0.95; 1.49)	1.19 (0.95; 1.48)	0.98 (0.73; 1.33)	0.99 (0.74; 1.34)	**1.65 (1.24; 2.19)**	**1.66 (1.26; 2.20)**	1.16 (0.90; 1.48)	1.14 (0.89;1.46)
Women	**1.41 (1.27; 1.56)**	**1.43 (1.29; 1.58)**	**1.31 (1.14; 1.50)**	**1.31 (1.15; 1.51)**	1.10 (0.96; 1.27)	1.09 (0.96;1.26)	1.07 (0.96; 1.19)	1.08 (0.97;1.20)
Age (years)	0.98 (0.97; 0.99)	0.99 (0.98; 0.99)	0.98 (0.97; 0.99)	0.99 (0.98; 1.00)	1.01 (1.00; 1.01)	1.01 (1.00;1.02)	**1.02 (1.01; 1.02)**	**1.03 (1.02;1.03)**
Number of medications	**1.10 (1.08; 1.13)**	-	**1.13 (1.10; 1.16)**	-	**1.07 (1.05; 1.10)**	-	**1.20 (1.17; 1.22)**	-
Medication with side-effects	-	**1.20 (1.05; 1.37)**	-	**1.58 (1.35; 1.85)**	-	**1.62 (1.39;1.98)**	-	**1.73 (1.52;1.96)**
Depression and/or anxiety (SHIP)	**3.45 (3.08; 3.86)**	**3.52 (3.15; 3.95)**	**8.14 (7.14; 9.28)**	**8.06 (7.06; 9.19)**	**7.03 (6.15; 8.04)**	**6.85 (5.99;7.84)**	**2.72 (2.41; 3.07)**	**2.74 (2.43; 3.09)**
Insomnia (SHIP)	**2.23 (2.00; 2.49)**	**2.31 (2.07; 2.57)**	**2.02 (1.76; 2.32)**	**2.09 (1.83; 2.40)**	**1.69 (1.48; 1.95)**	**1.72 (1.50; 1.97)**	**1.63 (1.45; 1.83)**	**1.72 (1.53; 1.93)**
CCI (claims data)	**1.11 (1.05; 1.18)**	**1.20 (1.14; 1.26)**	1.06 (0.98; 1.13)	**1.14 (1.07; 1.22)**	1.08 (1.02; 1.16)	**1.12 (1.05;1.19)**	**1.08 (1.02; 1.15)**	**1.19 (1.13; 1.26)**
Heart failure (claims data)	-	-	-	-	-	-	**2.72 (1.78; 4.16)**	**3.07 (2.03; 4.65)**
COPD (claims data)	-	-	-	-	-	-	**2.13 (1.59; 2.85)**	**2.29 (1.72; 3.06)**
Asthma (claims data)	-	-	-	-	-	-	**3.41 (2.69; 4.32)**	**3.66 (2.90; 4.62)**

OR: Odds Ratio, 95% CI: 95% Confidence Interval, CCI: Charlson-Comorbidity-Index, COPD: Chronic obstructive pulmonary disease, SHIP: Study of Health in Pomerania, bold numbers = statistically significant
